# Accuracy Verification of Spatio-Temporal and Kinematic Parameters for Gait Using Inertial Measurement Unit System

**DOI:** 10.3390/s20051343

**Published:** 2020-02-29

**Authors:** Sang Seok Yeo, Ga Young Park

**Affiliations:** 1 Department of Physical Therapy, College of Health Sciences, Dankook University, 119, Dandae-ro, Dongnam-gu, Cheonan-si, Chungnam 330-714, Korea; eangbul@hanmail.net; 2 Department of Physical Therapy, Graduate School, Dankook University, 119, Dandae-ro, Dongnam-gu, Cheonan-si, Chungnam 330-714, Korea

**Keywords:** gait, Spatio-temporal, kinematic, inertial measurement unit (IMU) system, optical motion capture (OMC) system

## Abstract

Inertial measurement unit systems are wearable sensors that can measure the movement of a human in real-time with relatively little space and high portability. The purpose of this study was to investigate the accuracy of the inertial measurement unit (IMU) system for gait analysis by comparing it with measurements obtained using an optical motion capture (OMC) system. To compare the accuracies of these two different motion capture systems, the Spatio-temporal and kinematic parameters were measured in young adults during normal walking. Thirty healthy participants participated in the study. Data were collected while walking 5 strides on a 7 m walkway at a self-selected speed. Results of gait analysis showed that the Spatio-temporal (stride time, stride length, cadence, step length) and kinematic (knee joint peak to peak of movement) parameters were not significantly different in the participant. Spatio-temporal and kinematic parameters of the two systems were compared using the Bland–Altman method. The results obtained showed that the measurements of Spatio-temporal and kinematic parameters of gait by the two systems were similar, which suggested that IMU and OMC systems could be used interchangeably for gait measurements. Therefore, gait analysis performed using the wearable IMU system might efficiently provide gait measurements and enable accurate analysis.

## 1. Introduction

Walking involves moving the body safely from one point to another through repeated, alternating movements of the lower extremities [1]. The initiation of walking is usually natural and does not require higher cognitive concentration in ordinary conditions [2]. The walking, which shows an automated movement pattern, is a combination of coordination and muscular activities and biomechanical forces [3]. Quality of walking refers to the ability of an individual to maintain safe walking at optimal energy costs while locomotion from varying external environmental variables [4]. On the other hand, muscular weakness, loss of sensation, severe pain, and motor control dysfunctions can impair gait function, and, importantly, patterns of gait disturbance indicate the patients’ problems and guide therapeutic intervention [5].

Gait analysis can objectively and quantitatively evaluate gait patterns associated with the functional limitations of individuals with a disability, and evaluations of Spatio-temporal gait parameters provide the most efficient means of gait and predicting fall risk [6,7]. Furthermore, the kinetic and kinematic variables of gait are also used to evaluate quantitatively the level of gait [8]. Optical motion capture (OMC) system, which utilizes multiple optical cameras to calculate 3D positions of body joints, is most commonly used to analyze gait phases and has the advantage of providing real-time measures of movement a rapid reaction rate and the ability to measure data close to the human movements even for complex motions [7,9].

Over the past few years, a number of wearable inertial measurement unit (IMU) systems have been used to analyze human joint movement in laboratories or in living environments [10]. The IMU system is useful because the system function is verified in the gait analysis of people with disabilities in low cost and non-invasive methods [11,12,13]. In order to increase the universal use of the IMU system, it is very necessary to analyze the accuracy of the IMU system by comparing the results of the gait analysis of the IMU system and the OMC system. Inertial measurement unit systems use accelerometers, gyroscopes, and magnetometers to provide data on the motion characteristics of joints and segments during various tasks [10]. IMU system can measure human joint movements in real-time and has the advantage of being able to measure in relatively little space and is highly portable compared to the OMC system. However, the disadvantage of the IMU system is that it includes a position drift that may vary over time with lower position accuracy. Position accuracy is a problem because it is determined by constantly integrating acceleration over time to calculate speeds and positions, and this results in cumulative measurement errors. In addition, since the position of the IMU sensors for measuring joint movement does not match the anatomical position of the actual joint, the corrected data must be converted into meaningful three-dimensional position data, such as a joint angle. For measuring the position data, the IMU system uses a similar analysis related to Wavelets to visualize gait identification [14,15,16]. Nevertheless, despite this shortcoming, the advantages of IMU systems are that they are increasingly utilized in industry and for medical diagnostic purposes [17,18]. Several previous studies of gait analysis reported the diagnostic significance of the IMU system by comparing the IMU system and the OMC system [10,19]. In particular, the significance of Spatio-temporal parameters is reported to be very high in gait analysis using the IMU system [2,4,20]. On the other hand, there are not many reports on the accuracy of gait kinematic parameters using the IMU system.

Therefore, the present study was undertaken to verify the accuracy of the inertial measurement unit system for gait analysis and to compare it with that of the optical motion capture system. To compare the accuracy of the two different motion capture systems, the Spatio-temporal and kinematic parameters were measured for young adults during normal gait.

## 2. Methods

Thirty healthy young adults (15 males, 15 females) participated in the study (Table 1). The study inclusion criteria were as follows: (1) no history of musculoskeletal or neurologic disease or cognitive problem and (2) no medical problem associated with the balance and gait.

All participants were provided explanations for the study and informed written consent. The study was approved beforehand by the institutional review board of the local University.

### 2.1. Measurement Equipment

#### 2.1.1. Gait Analysis Using the IMU System

**Figure 1:**
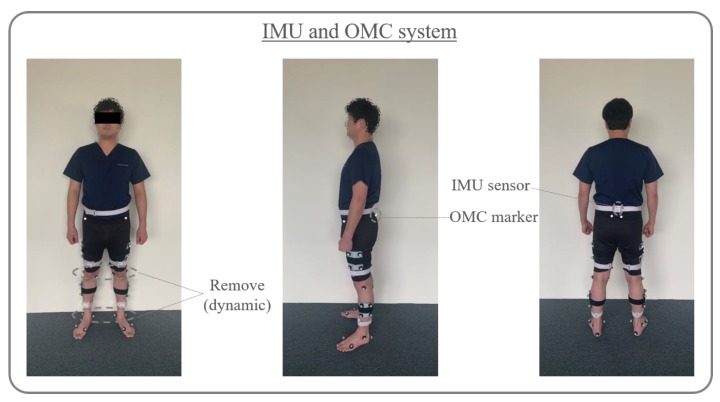
Sensor and marker positions in the inertial measurement unit (IMU) and optical motion capture (OMC) systems.

LEGSys+ wearable device (BioSensics, Cambridge, MA, USA) was used to measure Spatio-temporal and kinematic parameters of gait. Five sensors of LEGSys+ (5.0 cm × 4.2 cm × 1.2 cm) were connected to the computer via Bluetooth and included a 3-axis accelerometer, gyroscope, and magnetometer [21]. For the orientation of the sensor’s axis, the x-axis was set to a vertical direction, the y-axis was set to an antero-posterior direction, and the z-axis was set to a medio-lateral direction [22]. A sensor was attached to the center of the posterior superior iliac spine and the anterior surfaces of both shin (3 cm above the ankle) and thigh (3 cm above the knee) (Figure 1). The LEGSys sensors were composed of a triaxial accelerometer (±2 g) and a triaxial gyroscope (±2000 deg/s) [23]. The sampling rate of the sensors used 100 Hz [2].

#### 2.1.2. Gait Analysis Using the OMC System

This analysis was performed using the Qualisys system, which consisted of six infrared cameras acquiring the 3D coordinates of reflective markers (14 mm in diameter) affixed to participants using Qualisys track manager (QTM) motion capture. For the orientation of the sensor’s axis, the x-axis was set to a medio-lateral direction, the y-axis was set to an antero-posterior direction, and the z-axis was set to a vertical direction. The 3D coordinates of the markers were filtered through a low-pass Butter–worth digital filter at a cut-off frequency of 6 Hz [24]. The marker attachment site of the Qualisys motion capture system was a Coda marker set. A total of 34 markers were attached to the pelvis, thigh, knee, calf, ankle, heel, and 1st and 5th toes during static measurement. For dynamic measurement, knee and ankle markers were removed, and 30 markers were attached (Figure 1). The sampling rate of the Qualisys system used in the present study was 100 Hz [20,25].

#### 2.1.3. Gait Analysis Using the IMU System

**Figure 2:**
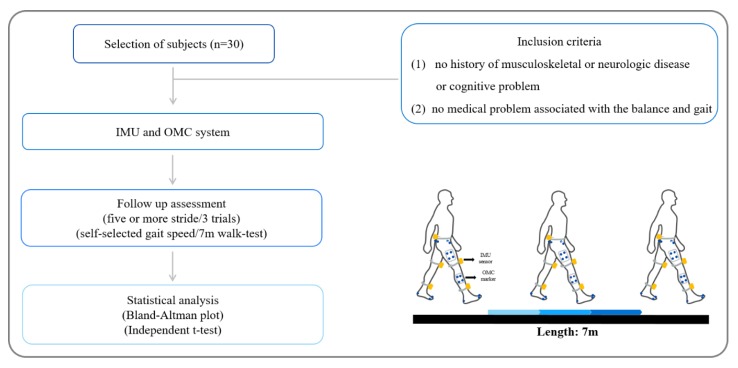
Schematic diagram of gait analysis using the IMU system and OMC system.

Data was collected using the LEGSys+ system with five or more strides along 7 m walkway at self-selection speed. Spatio-temporal and kinematical data were recorded for the middle three strides (Figure 2). A total of three attempts were made, and the rest was necessary. The study analyzed the one-stride’s time and length, cadence, length of left and right step, and the range of motion of knee and hip joint during gait [26]. The range of knee and hip joint was calculated from the maximum angle of each subject minus the minimum angle. The time to each peak of the joint was expressed as a percentage of total gait cycle duration [27]. The Spatio-temporal and kinematic data were collected for gait analysis using the Biosensics software program (LEGSys; BioSensics, Cambridge, MA, USA) [21,28]. The reflective markers of the OMC system were used simultaneously to assess the accuracy of the IMU.

#### 2.1.4. Gait Analysis Using the OMC System

Qualisys’ gait measurements were measured in the space of 9.2 m³ (2 m × 2 m × 2.3 m). Static data were collected in an anatomical posture, and 3D dynamic gait analysis data were processed for each participant. Data was collected using the Qualisys system with five or more strides along 7 m walkway at self-selection speed. Spatio-temporal and kinematical data were recorded for the middle three strides (Figure 2). A total of three dynamic gait analyses were collected and allowed to rest between gait measurements if necessary. Spatio-temporal and kinematic data were collected for the middle three steps. The study analyzed one-stride’s time and length, cadence, length of left and right step, and the range of motion of knee and hip joint during gait. The range of knee and hip joint was calculated from the maximum angle of each subject minus the minimum angle [27]. The time to each peak of the joint was expressed as a percentage of total gait cycle duration. Kinematic gait data collected by the infrared camera were quantified using the Visual 3D motion analysis program (C–Motion, Rockville, MD, USA) [29]. The reflective markers of the OMC system were used simultaneously to assess the accuracy of the IMU.

### 2.2. Statistical Analysis

SPSS 21.0 for the window (IBM Inc, Chicago, IL, USA) was used for statistical analysis of the experiment. Bland–Altman plots were used to compare the coincidence levels of the IMU and OMC systems with respect to gait measurement data. Bland-Altman plots allow comparisons between two different measurement systems when evaluating the same dataset to evaluate the match level [30]. Independent sample t-tests were also used to compare the mean differences between the two systems. The statistically significant p-value was set at 0.05.

## 3. Results

Gait parameters, as measured by the IMU and OMC systems, are shown in Table 2. There was no significant difference in the average comparison of Spatio-temporal parameters between two systems (*p* > 0.05) (Table 2). Regarding kinematic parameters, both knee joint movements were not significantly different (*p* > 0.05) (Table 3), but the IMU system showed a significantly higher movement range of hip joint compared with the OMC system (*p* < 0.05). 

**Figure 3:**
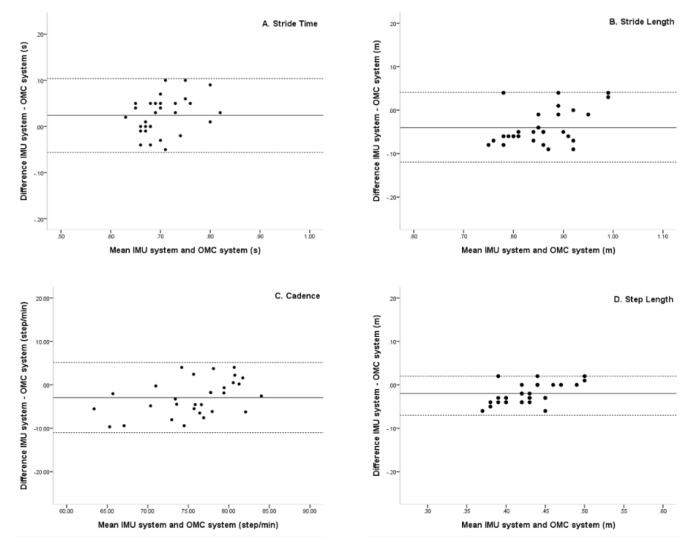
Bland–Altman plots comparing IMU system (LEGSys+) and OMC system (Qualisys) results for (**A**) Stride time, (**B**) Stride length, (**C**) Cadence, and (**D**) Step length left and right. Bias (solid line) and limits of agreement are (dashed line) shown for each variable. The mean score is plotted on the x-axis, and the difference between the two devices is plotted on the y-axis (mean difference ± 1.96 SD).

Mean differences between the IMU and OMC systems for stride time, stride length, cadence, and step length were 0.02, −0.04, −2.92, and −0.02, respectively. In Bland–Altman plots, the limit of agreement for stride time, stride length, cadence, and step length were 0.10 to −0.06, 0.04 to −0.12, 5.14 to −10.98, and 0.02 to −0.07, respectively. All Spatio-temporal parameters were within a 95% limit of agreement from the means of differences between the IMU and OMC systems (Figure 3).

**Figure 4:**
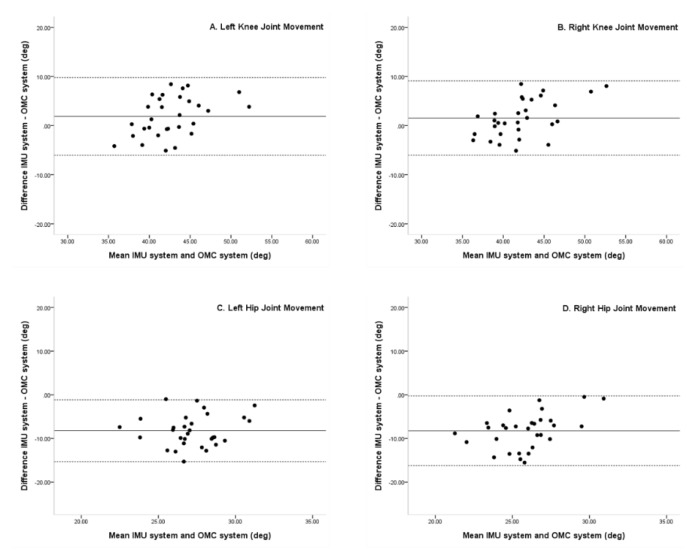
Bland–Altman plots comparing IMU system (LEGSys+) and OMC system (Qualisys) results for peak to peak (**A**) Left knee joint degree, (**B**) Right knee joint degree, (**C**) Left hip joint degree, and (**D**) Right hip joint degree. Bias (solid line) and limits of agreement (dashed line) are shown for each variable. The mean score is plotted on the x-axis, and the difference between the two devices is plotted on the y-axis (mean difference ± 1.96 SD).

Mean differences between the knee and hip joint movements were 1.87, 1.54, −8.21, and −8.25, left and right, respectively. In Bland–Altman plots, the limit of agreement in the knee and hip joint movements was 9.78 to −6.03, 9.10 to −6.02, −1.1 to −15.31, and −0.25 to −16.25, left and right, respectively. In addition, Bland–Altman plots showed knee and hip joint movements to be within 95% limit of agreement for the IMU and OMC system (Figure 4).

## 4. Discussion

In the current study, we sought to verify the accuracy of the IMU system in young adults during normal gait by comparing measurements with the OMC system. We found no significant differences in the Spatio-temporal parameters between the two systems. In the kinematic parameters of the two systems, there was no significant difference in the knee joint movements of both the systems; however, there was a significant difference in the hip joint movements of the systems. In contrast, the result of Bland–Altman plots showed that Spatio-temporal and kinematic parameters were within a 95% limit of agreement. The x-axis of the Bland–Altman plot was the average of each value between the IMU and OMC systems, and the y-axis was the difference between the two systems. The match level was high because the mean of the differences between A, B, C, and D was located on the upper and lower limits of agreement. The mean difference indicated whether there was a bias between the two methods and whether one method tended to overestimate or underestimate on average compared to the other. The closer the mean difference to zero is obtained from the two gait analysis system, the higher is the agreement and the two systems are interchangeable [30,31].

In a number of previous studies, the OMC system has been used to assess gait abilities in normal adults or patient groups. Studies on normal adults have mainly focused on age-related changes in gait parameters. Ko et al. used an OMC system to investigate age-related changes in gait parameters in 190 healthy adults aged from 32 to 93 years and stated the advantages of OMC system that it could objectively and quantitatively determine age-related reductions in ROM of ankle, knee, and hip joints and in walking speed due to aging [24]. On the other hand, there is a study that examines how the elements of gait deviation during gait analysis influences clinical decision-making for a patient. The authors suggested that attaching pelvis markers lower than the criterion was likely to reduce measured reductions in anterior and posterior pelvic tilt angles during gait. Therefore, it is necessary to identify and correct the experimental deviation and to critically evaluate the deviation according to the position of the marker. In addition, it has also been reported that inaccurate marker placement at the anterior superior iliac spines (ASIS) and posterior superior iliac spines (PSIS) can cause hip joint curve errors [32].

Knee and hip joint angles measured over gait cycles as determined by the IMU and OMC systems seemed to follow the same trends, but magnitudes differed substantially. Xu et al. reported that wearable sensor system did not detect variabilities in pelvis anterior-posterior tilt and reported that wearable sensors were able to determine approximate joint trajectory, but could not measure fine changes in joint angles [33]. Wu et al. reported pelvic tilt was related to the cephalocaudal position between ASIS and PSIS. In addition, due to the characteristics of the wearable sensor system, joint angles were measured using an algorithm calculated through the position between the sensors without directly attaching a marker and a position drift that might change with time, thereby causing an error [34].

Recently, research on gait analysis performed using a wearable IMU system has been actively conducted. Seel et al. reported an average knee joint angle error of less than about 3° and an average ankle joint angle error of less than about 1° in a study on the use of a wearable inertial sensor system to measure joint angles for gait analysis [18]. Washabaugh et al. reported on the effectiveness and repeatability of a wearable inertial sensor system for the measurement of gait patterns variability and showed the effectiveness of the system for measuring Spatio-temporal parameters, such as gait speed, stride length, speed, posture ratio, and gait cycle, in 39 healthy young subjects. This previous study was conducted using a standard treadmill, a split-belt treadmill, and over the ground, and gait pattern data measured by the IMU system were verified using standard clinical procedures. The authors suggested that gait analysis using the IMU system offered a suitable means of accurately measuring Spatio-temporal parameters of gait pattern [17].

In addition, two previous studies compared the performances of IMU and OMC systems for gait analysis. Lanovaz et al. examined 10 children to determine whether an IMU system was appropriate for gait evaluation by comparing results with those of an OMC system [10]. The authors concluded that the two measurement methods showed high levels of agreement for step time, step length, step speed, and step time. Subsequently, Lee et al. evaluated the validities of the IMU and OMC methods for evaluating gait in 17 Parkinson’s disease patients and found a high level of agreement for cadence, step length, and step time [19]. However, in these studies, only Spatio-temporal parameters were reported, and kinematic variables were not compared.

The present study has a number of limitations that warrant consideration. First, it is difficult to generalize our results because of the small sample size. Second, the study participants consisted of young adults in their 20′s. Accordingly, care should be taken when interpreting our results. It is necessary to choose a way to reduce the error by recruiting a larger number of samples, including different age groups. Third, ankle joint angles were not included due to a lack of sensors for the IMU system. Lastly, the present study was measured in a limited laboratory setting for measuring the OMC system; it is necessary to be measured under various conditions, such as different floor or obstacles. Further studies should be conducted to verify the reliability and validity of the IMU system for measuring Spatio-temporal and kinematic parameters. 

## 5. Conclusions

In this study, we compared gait measurements obtained using an optical motion capture system and a wearable inertial measurement unit system. In order to verify the accuracy of the IMU system, we compared the level of agreement between Spatio-temporal and kinematic measurements in normal young adults during walking. Comparisons of the measurements using Bland–Altman plot showed that average Spatio-temporal and kinematic variable measurements obtained using the IMU and OMC systems were in the 95% limit of agreement (LOA), and the level was high. These results indicated that the two systems could be used interchangeably in gait measurements. Therefore, gait analysis using wearable inertial measurement unit system might be helpful for efficient and accurate measurements of gait analysis.

## Figures and Tables

**Table 1:** General characteristics of subjects.

	Number of Subjects	Age	Height (cm)	Weight (kg)
Male	15	24.47(2.53)	176.53(4.88)	72.8(8.27)
Female	15	22.53(0.74)	162.33(4.43)	57.33(5.77)

Values represent mean (± standard deviation).

**Table 2:** Spatio-temporal parameters during gait in the IMU system and OMC system.

	Stride Time (s)	Stride Length (m)	Cadence (step/min)	Step Length (m)
IMU system	1.03	1.33	116.94	0.67
	(0.07)	(0.84)	(7.45)	(0.04)
OMC system	1.06	1.29	113.45	0.64
	(0.07)	(0.96)	(7.92)	(0.05)
t	1.39	−1.71	−1.76	−1.89
*p*	0.17	0.09	0.08	0.06

Values represent mean (±standard deviation). IMU: inertial measurement unit system; OMC: optical motion capture. * *p* < 0.05.

**Table 3:** Kinematic parameters during gait in the IMU system and OMC system.

	Knee Joint (Peak to Peak)		Hip Joint (Peak to Peak)	
	Left	Right	Left	Right
IMU system	62.13	61.76	49.02	47.05
	(5.18)	(4.92)	(4.38)	(3.92)
OMC system	64.00	63.30	40.81	38.81
	(5.37)	(5.76)	(2.96)	(3.17)
t	1.38	1.11	−8.50	−8.97
*p*	0.17	0.27	0^*^	0^*^

Values represent mean (±standard deviation). IMU: inertial measurement unit system; OMC: optical motion capture. * *p* < 0.05.

## References

[1] Vachranukunkiet T., Esquenazi A. (2013). Pathophysiology of gait disturbance in neurologic disorders and clinical presentations. Phys. Med. Rehabil. Clin. N. Am..

[2] Yeo S.S., Cho I.H. (2018). Gait Characteristic in a Stroke Patient with an Intact Corticospinal Tract and Corticoreticular Pathway: A Case Study. J. Korean Phys. Ther..

[3] Esquenazi A. (2014). Gait analysis in lower-limb amputation and prosthetic rehabilitation. Phys. Med. Rehabil. Clin. N. Am..

[4] Najafi B., Khan T., Wrobel J. Laboratory in a box: Wearable sensors and its advantages for gait analysis. Proceedings of the 2011 Annual International Conference of the IEEE Engineering in Medicine and Biology Society.

[5] Burnfield M. (2010). Gait analysis: Normal and pathological function. J. Sports Sci. Med..

[6] Donath L., Faude O., Lichtenstein E., Nuesch C., Mundermann A. (2016). Validity and reliability of a portable gait analysis system for measuring spatiotemporal gait characteristics: Comparison to an instrumented treadmill. J. Neuroeng. Rehabil..

[7] Gomez Bernal A., Becerro-de-Bengoa-Vallejo R., Losa-Iglesias M.E. (2016). Reliability of the OptoGait portable photoelectric cell system for the quantification of spatial-temporal parameters of gait in young adults. Gait Posture.

[8] Tao W., Liu T., Zheng R., Feng H. (2012). Gait analysis using wearable sensors. Sensors.

[9] Liu T., Inoue Y., Shibata K. (2009). Development of a wearable sensor system for quantitative gait analysis. Measurement.

[10] Lanovaz J.L., Oates A.R., Treen T.T., Unger J., Musselman K.E. (2017). Validation of a commercial inertial sensor system for spatiotemporal gait measurements in children. Gait Posture.

[11] Sijobert B., Denys J., Coste C.A., Geny C. IMU based detection of freezing of gait and festination in Parkinson’s disease. Proceedings of the 2014 IEEE 19th International Functional Electrical Stimulation Society Annual Conference (IFESS).

[12] Margiotta N., Avitabile G., Coviello G. A wearable wireless system for gait analysis for early diagnosis of Alzheimer and Parkinson disease. Proceedings of the 2016 5th International Conference on Electronic Devices, Systems and Applications (ICEDSA).

[13] Cando O.A., Hidalgo K.R., Palacios B.C. A low-cost vibratory stimulus system to mitigate freezing of gait in Parkinson’s disease. Proceedings of the 2016 IEEE ANDESCON.

[14] Glowinski S., Blazejewski A., Krzyzynski T. (2017). Inertial sensors and wavelets analysis as a tool for pathological gait identification. Innovations in Biomedical Engineering.

[15] Glowinski S., Blazejewski A., Krzyzynski T. (2017). Human gait feature detection using inertial sensors wavelets. Wearable Robotics: Challenges and Trends.

[16] Najafi B., Lee-Eng J., Wrobel J.S., Goebel R. (2015). Estimation of center of mass trajectory using wearable sensors during golf swing. J. Sports Sci. Med..

[17] Washabaugh E.P., Kalyanaraman T., Adamczyk P.G., Claflin E.S., Krishnan C. (2017). Validity and repeatability of inertial measurement units for measuring gait parameters. Gait Posture.

[18] Seel T., Raisch J., Schauer T. (2014). IMU-based joint angle measurement for gait analysis. Sensors.

[19] Lee M., Youm C., Jeon J., Cheon S.M., Park H. (2018). Validity of shoe-type inertial measurement units for Parkinson’s disease patients during treadmill walking. J. Neuroeng. Rehabil..

[20] Yang S., Zhang J.-T., Novak A.C., Brouwer B., Li Q. (2013). Estimation of spatio-temporal parameters for post-stroke hemiparetic gait using inertial sensors. Gait Posture.

[21] Najafi B., Helbostad J.L., Moe-Nilssen R., Zijlstra W., Aminian K. (2009). Does walking strategy in older people change as a function of walking distance?. Gait Posture.

[22] Hsu W.-C., Sugiarto T., Lin Y.-J., Yang F.-C., Lin Z.-Y., Sun C.-T., Hsu C.-L., Chou K.-N.J.S. (2018). Multiple-Wearable-Sensor-Based Gait Classification and Analysis in Patients with Neurological Disorders. Sensors.

[23] Zhou H., Al-Ali F., Kang G., Hamad A., Ibrahim R., Talal T., Najafi B. (2020). Application of Wearables to Improve Uptake of Exercise Therapy during Hemodialysis Treatment for Reducing Depression Symptom—A Single Blinded Randomized Controlled Trial. Med. Pharmacol..

[24] King S.L., Barton G.J., Ranganath L.R. (2017). Interpreting sources of variation in clinical gait analysis: A case study. Gait Posture.

[25] McGinley J.L., Baker R., Wolfe R., Morris M.E. (2009). The reliability of three-dimensional kinematic gait measurements: A systematic review. Gait Posture.

[26] Gill S.V., Walsh M.K., Pratt J.A., Toosizadeh N., Najafi B., Travison T.G. (2016). Changes in spatiotemporal gait patterns during flat ground walking and obstacle crossing 1 year after bariatric surgery. Surg. Obes. Relat. Dis..

[27] Chen C.L., Chen H.C., Tang S.F., Wu C.Y., Cheng P.T., Hong W.H. (2003). Gait performance with compensatory adaptations in stroke patients with different degrees of motor recovery. Am. J. Phys. Med. Rehabil..

[28] Muchna A., Najafi B., Wendel C.S., Schwenk M., Armstrong D.G., Mohler J.J. (2018). Foot problems in older adults: Associations with incident falls, frailty syndrome, and sensor-derived gait, balance, and physical activity measures. J. Am. Podiatr. Med. Assoc..

[29] Weidow J., Tranberg R., Saari T., Karrholm J. (2006). Hip and knee joint rotations differ between patients with medial and lateral knee osteoarthritis: Gait analysis of 30 patients and 15 controls. J. Orthop. Res..

[30] Bland J.M., Altman D.G. (2003). Applying the right statistics: Analyses of measurement studies. Ultrasound Obstet. Gynecol..

[31] Bartlett J., Frost C. (2008). Reliability, repeatability and reproducibility: Analysis of measurement errors in continuous variables. Ultrasound Obstet. Gynecol..

[32] Ko S.U., Stenholm S., Metter E.J., Ferrucci L. (2012). Age-associated gait patterns and the role of lower extremity strength-results from the Baltimore Longitudinal Study of Aging. Arch. Gerontol. Geriatr..

[33] Xu X., McGorry R.W., Chou L.S., Lin J.H., Chang C.C. (2015). Accuracy of the Microsoft Kinect for measuring gait parameters during treadmill walking. Gait Posture.

[34] Wu G., Siegler S., Allard P., Kirtley C., Leardini A., Rosenbaum D., Whittle M., D’Lima D., Cristofolini L., Witte H. (2002). ISB recommendation on definitions of joint coordinate system of various joints for the reporting of human joint motion—Part I: Ankle, hip, and spine. J. Biomech..

